# Microbubble-Mediated Ultrasound Enhances the Lethal Effect of Gentamicin on Planktonic *Escherichia coli*

**DOI:** 10.1155/2014/142168

**Published:** 2014-05-15

**Authors:** Han-Xiao Zhu, Xun-Zi Cai, Zhong-Li Shi, Bin Hu, Shi-Gui Yan

**Affiliations:** Department of Orthopaedic Surgery, Second Affiliated Hospital, School of Medicine, Zhejiang University, No. 88 Jiefang Road, Hangzhou 310009, China

## Abstract

Previous research has found that low-intensity ultrasound enhanced the lethal effect of gentamicin on planktonic *E. coli*. We aimed to further investigate whether microbubble-mediated low-intensity ultrasound could further enhance the antimicrobial efficacy of gentamicin. The planktonic *E. coli* (ATCC 25922) was distributed to four different interventions: control (G_CON_), microbubble only (G_MB_), ultrasound only (G_US_), and microbubble-mediated ultrasound (G_MUS_). Ultrasound was applied with 100 mW/cm^2^ (average intensity) and 46.5 KHz, which presented no bactericidal activity. After 12 h, plate counting was used to estimate the number of bacteria, and bacterial micromorphology was observed with transmission electron microscope. The results showed that the viable counts of *E. coli* in G_MUS_ were decreased by 1.01 to 1.42 log_10_ CFU/mL compared with G_US_ (*P* < 0.01). The minimal inhibitory concentration (MIC) of gentamicin against *E. coli* was 1 **μ**g/mL in the G_MUS_ and G_US_ groups, lower than that in the G_CON_ and G_MB_ groups (2 **μ**g/mL). Transmission electron microscopy (TEM) images exhibited more destruction and higher thickness of bacterial cell membranes in the G_MUS_ than those in other groups. The reason might be the increased permeability of cell membranes for gentamicin caused by acoustic cavitation.

## 1. Introduction

Antibiotics are commonly prescribed to cure diseases from epifolliculitis to fatal infections. However, bacteria are becoming increasingly resistant to antibiotics. In Europe, more than 25,000 patients die every year from infectious diseases because of multiresistant bacteria [1]. Antibiotic resistance is caused by many mechanisms, including reduced bacterial membrane permeability and gene mutations which change the targets of antibiotics and produce efflux proteins which pump the antibiotic out of the bacteria [2].

A series of literatures have found that low-intensity ultrasound enhances gentamicin killing of planktonic* E. coli,* while ultrasound alone does not kill bacteria [3–7]. It is suggested that stable cavitation of ultrasound might contribute to altering structure of bacterial cell membrane, thus facilitating the penetration of bacteria by antibiotics [8]. Our laboratory has found that low-intensity ultrasound enhances the antimicrobial efficacy of vancomycin against* S. aureus* in bone cements [9, 10]. Low-intensity ultrasound shows promise for enhancement of antibiotics actions for its easy access, noninvasiveness, and safety [2].

Recently microbubble-mediated ultrasound (MUS) has been increasingly used to improve the therapeutic effects of ultrasound in the fields of transdermal drug delivery, thrombolysis, and transfection of gene vectors [11, 12]. Microbubbles have a gas-filled structure, stabilized by a protein, lipid, or polymer shell; some microbubbles have been clinically approved as contrast agent. MBs can provide nuclei and lower the threshold for cavitation during sonication. Whether MUS enhances the bactericidal effect of gentamicin more than ultrasound (US) does is unknown. Therefore, we designed this study to investigate the* in vitro* response of planktonic* E. coli* to the combination of MUS and gentamicin.

## 2. Materials and Methods

### 2.1. Organisms and Antibiotic

Culture of* Escherichia coli *(ATCC 25922) was selected for this study, which was maintained on blood agar plates. 24 h before an experiment, an inoculum was transferred from a colony on the agar plate to 10 mL of tryptic soy broth (TSB) without glucose (Oxoid, Basing stoke, UK) and grown overnight at 37°C. After 24 h, 0.01 mL of the bacteria fluid was transferred to 9.99 mL of sterile TSB on a rotary shaker and grown at 37°C. Growth curves showed that the bacteria were in the exponential phase of growth between one and ten hours. The number of bacteria in the suspensions was measured by serial dilutions in physiological saline solution (PSS) and plating onto nutrient agar. Plates were incubated for 24 h at 37°C. Gentamicin sulfate (Sigma, St. Louis, MO, USA) was reconstituted in distilled water without further purification.

### 2.2. MB and Ultrasound

**Figure 1:**
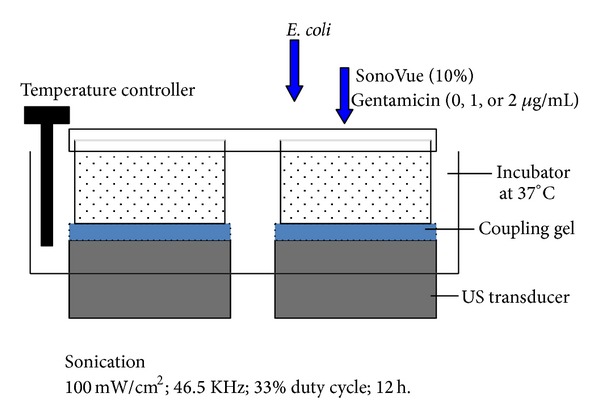
Experimental design. Schematic drawing of the ultrasound setting.* E. coli* was exposed to ultrasound after addition of gentamicin and SonoVue (microbubble).

Figure 1 shows an illustration of the experimental apparatus for ultrasonic exposure. Sonovue as MB contrast agent (Bracco, Milan, Italy) was used in this study. The diameter of MB is typically 1 to 8**μ**m (mean, 2.5 *μ*m) [13]. The shell of MB is composed of phospholipids and encapsulates sulfur hexafluoride gas. The MB was reconstituted in 5 mL normal saline and the solution contained 2–5 × 10^8^ MBs/mL. The concentration of the initial solution was defined as 100% and the MB solution was further diluted in the TSB medium (1 : 9) in the MB only and MUS experiments; therefore, the final concentration was defined as 10% (*v/v*).

The ultrasonic generator (Nexus; Hexin Biomedical Devices, Hangzhou, China) was employed in these experiments with four ultrasonic transducers (operating at 46.5 KHz) in a bath. The bath was filled with water and maintained at 37°C. The temperature of the bacterial suspensions inside the tubes was monitored. Both the intensity (100 mW/cm^2^) as well as the frequency was calibrated by the manufacturer. The duty cycle was 1 : 3.

### 2.3. Measurement of MIC

The minimum inhibitory concentration (MIC) of gentamicin was determined as reported previously [3]. The MIC was measured by preparing a series of test tubes containing TSB and gentamicin at concentrations between 0 and 4 *μ*g/mL (in 1 *μ*g/mL increments). Each tubewas inoculated with exponential growth-phase* E. coli* and incubated at 37°C for 24 h. Then, the concentrations of the cultures were measured by plate counting and the turbidities of the tubes were assessed. The MIC was defined as the lowest concentration of gentamicin that had viable counts of less than 10^7^ CFU/mL. The MIC for* E. coli* in this study was 2 *μ*g/mL.

### 2.4. Bactericidal Activity

Frozen stocks were aerobically cultured onto blood agar plates at 37°C for 24 h. The bacterial suspension containing 10^7^ CFU/mL was prepared. 4 mL of bacteria suspension was placed into each tube. 0.5 mL of gentamicin or physiologic saline solution (PSS) was added to produce concentrations of gentamicin at 0, 1, or 2 *μ*g/mL. And 0.5 mL of MB solution or PSS was added to produce concentrations of MB at 0% or 10% (*v/v*). The planktonic* E. coli* was divided into control group (G_CON_), MB only group (G_MB_), US only group (G_US_), and MB + US group (G_MUS_). And there were 3 subunits (*n* = 8) in each group according to the gentamicin concentrations of 0, 1, or 2 *μ*g/mL. The tubes were placed in the bath. The ultrasonic transducers were placed 2 mm below each tube, and US was transmitted through the bottom of the tubes via a coupling gel. During experiments, the acoustic intensity (Average intensity) was set at 100 mW/cm^2^. After 12 h of an experiment, samples were taken from each tube, serially diluted in PSS, and 100 *μ*L was inoculated onto petri dishes containing nutrient agar by using the spread plate method. The petri dishes were incubated at 37°C for 24 h and counted. Parallel and identical sets of cultures were prepared at the time gentamicin and MB were introduced. The sets of US and MUS groups were placed in the sonicating bath, and the other sets were placed in an incubator. The mean and 95% confidence intervals of the log of the counts of colony forming units (CFU) per mL were calculated from the results of several replicate experiments.

### 2.5. Transmission Electron Microscope

After measurement of bactericidal activity, bacterial suspensions were transferred to examine ultrastructure of the bacteria. Histological assessment was conducted by electron microscopy. Bacteria suspensions were centrifuged at 5,000 rpm for 10 minutes. The liquid supernatant was removed and 4% glutaraldehyde was added on the pellet for fixation. The pellet was resuspended and stored at 4°C. Then, the bacteria were scanned by transmission electron microscope (JEM-1230, JOEL, Tokyo, Japan) to observe the microstructure. In order to quantify the alteration of bacterial wall, the thickness of bacterial cell wall was measured by using image analysis software (Image-Pro Plus 6.0, Media Cybernetics Inc., Silver Spring, MA, USA).

### 2.6. Statistical Analysis

The data of viable counts and thickness of cell wall were expressed as mean ± standard deviation. After Kolmogorov-Smirnov test for Gaussian distribution and a homogeneity test for variance were both passed, intergroup differences were compared by one-way analysis of variance (ANOVA) followed by the LSD post hoc test using SPSS version 16.0 (SPSS Inc., Chicago, IL, USA). A *P* value of <0.05 was considered statistical significant.

## 3. Results

### 3.1. Acoustically Enhanced Bactericides

**Figure 2:**
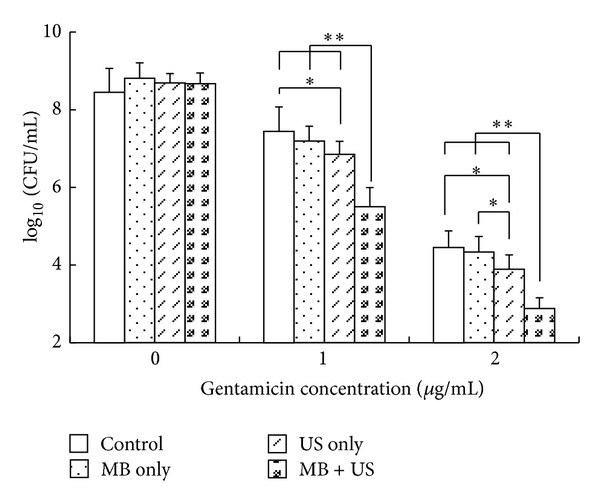
Comparison of viable bacteria recovered after sonication. The *x*-axis represents the concentration of gentamicin and the *y*-axis is the* E. coli* concentration of CFU. The three sets of bars show the viable counts at different concentrations of gentamicin without US and MB or with different combinations thereof. These data were expressed as mean ± standard deviation (*n* = 8). The error bars represent 95% confidence intervals. *P < 0.05, **P < 0.01.

Figure 2 shows the mean viability (95% confidence intervals) of* E. coli* after 12 h of sonication. When no gentamicin (0 *μ*g/mL) was added, there were no significant differences (*F* = 1.181,   *P* = 0.335) between the G_CON_ (8.45 ± 0.51) and the other three groups (G_MB_ 8.81 ± 0.40, G_US_ 8.67 ± 0.24, and G_MUS_ 8.69 ± 0.28). Without gentamicin, MB and low-intensity ultrasound were apparently insufficient to kill bacteria. At a gentamicin concentration of 1 *μ*g/mL, the* E. coli* concentration in the G_US_ (6.86 ± 0.29) was significantly lower than that in the G_CON_ (7.44 ± 0.64,  *P* < 0.05); and G_MUS_ was decreased to 5.44 ± 0.49, 1.42 log_10_ CFU/mL (*P* < 0.01) less than the* E. coli* concentration of the G_US_ groups. Both G_CON_ and G_MB_ (7.19 ± 0.38) groups contained more than 10^7^ CFU/mL and there was no significant difference (*P* = 0.304) between them. By contrast, both the G_US_ and G_MUS_ groups had viable counts of less than 10^7^ CFU/mL, which indicated that the MIC decreased from 2 *μ*g/mL to 1 *μ*g/mL in the presence of ultrasound and/or microbubble.

At a gentamicin concentration of 2 *μ*g/mL, the* E. coli* concentration in the G_US_ (3.89 ± 0.37) was lower than that in the G_CON_ (4.45 ± 0.49,  *P* < 0.05) and G_MB_ (4.33 ± 0.46,  *P* < 0.05) groups. Viable counts in the G_MUS_ (2.88 ± 0.28) were further decreased by 1.01 log_10_ CFU/mL (*P* < 0.01) compared with the G_US_ groups. The external microbubble (SonoVue) amplified the synergistic effect between ultrasound and antibiotics, which was observed in groups with gentamicin concentrations at 1 *μ*g/mL and 2 *μ*g/mL.

### 3.2. Microstructure of Bacteria

**Figure 3:**
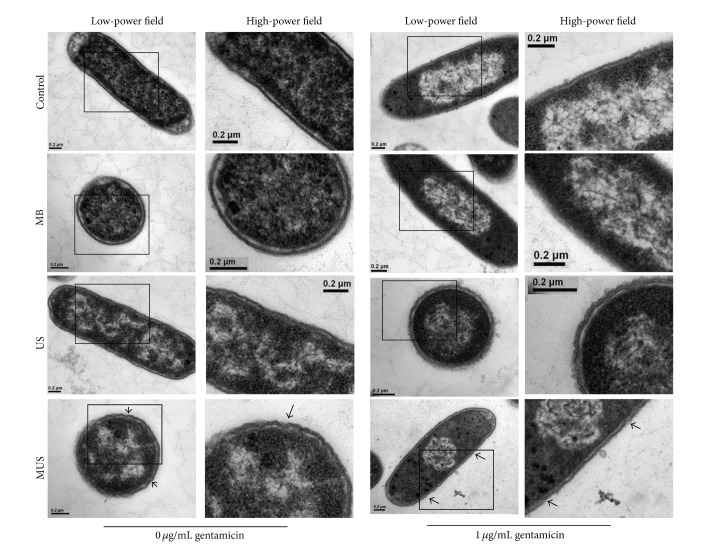
Structural images of bacteria. The structure of the bacteria of G_CON_ and G_MB_ groups was normal. The bacteria cell walls of G_US_ and G_MUS_ were wrinkled and thicker than those of G_CON_ and G_MB_. Partial destruction (arrows) could be seen in the bacterial cell walls in the G_MUS_ groups.

**Figure 4:**
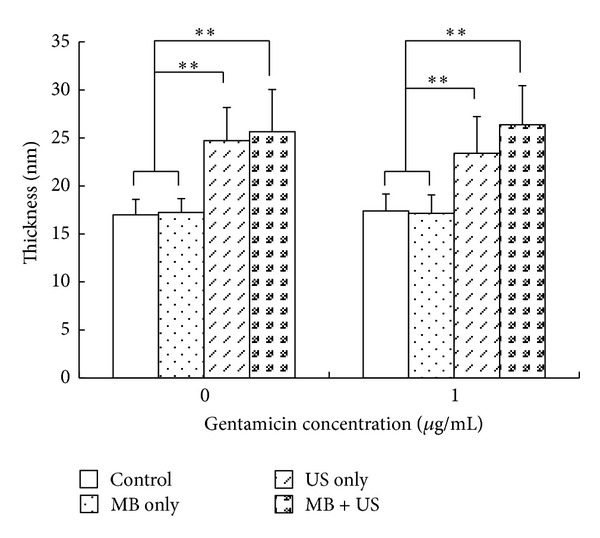
The thickness of bacterial cell wall. At both concentrations (0 *μ*g/mL and 1 *μ*g/mL), the mean thickness of bacterial cell walls of G_US_ and G_MUS_ groups was greater than that in the G_CON_ and G_MB_ groups (*P* < 0.01). Asterisks denote statistically significant differences; ^∗^ *P* < 0.05,   **P < 0.01.

Figure 3 shows pictures of the ultrastructure of* E. coli*. At gentamicin concentration of 2 *μ*g/mL, no pellet formation took place because of the low viable bacterial concentration. Therefore, only the bacteria exposed to gentamicin concentrations of 0 *μ*g/mL and 1 *μ*g/mL were examined by electron microscopy. In the G_CON_ and G_MB_ groups, the bacteria cell membranes were well preserved. In G_US_ and G_MUS_, partial destruction or disintegration of the cell membrane was detected.  Figure 4 showed the mean thickness (nm) of bacteria cell walls in each group. When the gentamicin concentration was 0 *μ*g/mL, the thickness of cell membranes in the G_US_ (24.38 ± 2.57) and G_MUS_ (25.31 ± 3.83) groups was significantly higher than that in the G_CON_ (17.00 ± 1.60, each *P* < 0.01) and G_MB_ groups (17.24 ± 1.43, each *P* < 0.01). At a gentamicin concentration of 1 *μ*g/mL, the thickness of cell membranes in the G_US_ (23.39 ± 3.83) and G_MUS_ (26.10 ± 4.08) groups was significantly higher than that in the G_CON_ (17.40 ± 1.74, each *P* < 0.01) and G_MB_ groups (17.14 ± 1.93, each *P* < 0.01). There was no significant difference between G_US_ and G_MUS_ groups in either of the two gentamicin concentrations: neither at 0 *μ*g/mL (*P* = 0.658) nor at 1 *μ*g/mL (*P* = 0.074).

## 4. Discussion

To our knowledge, the present study was the first to investigate the synergism of MUS and gentamicin regarding their lethal effect on planktonic* E. coli. *Our data indicated that MUS further enhanced the bactericidal effect of gentamicin on planktonic* E. coli* by more than one order of magnitude, compared with US while MUS alone did not reduce viability of bacteria. Furthermore, TEM images showed the partial destruction and greater thickness of the bacteria cell wall of G_US_ and G_MUS_, probably an explanation of the enhancing effect of MUS.

Advances in technologies have made US safely applicable for therapeutic purposes. Previous studies have shown synergism between US and antibiotics regarding their bactericidal effects (US alone is not bactericidal) [3, 4, 7, 8]. The mechanism of the synergism is complex. Runyan et al. argue that US could increase the permeability of bacterial cell membranes [14]. The term “sonoporation” is introduced to describe the temporal change in cell membrane permeability mediated by US [15, 16]. This change is successfully visualized in eukaryotic cell (HL-60) with electron microscope [17]. At a high acoustic pressure (>150 mW/cm^2^), transient cavitation leads to microbubble implosion [14]. At a low acoustic pressure, however, microbubbles in medium oscillate in a symmetrical way, known as stable cavitation. Such stable oscillation creates liquid flow around the microbubbles, which is called microstream. When the oscillating microbubble is in close vicinity of the cell, the shear stress can cause pore formation on the cell membrane. In our study, the low intensity (average of 100 mW/cm^2^) mainly induced stable cavitation. The phenomenon of sonoporation may contribute to the penetration of the bacterial cell membrane by antibiotics.

However, the occurrence of sonoporation on bacteria cell walls needs to be substantiated by solid evidence, because bacteria are much smaller and have a higher tensile strength than eukaryotic cell. Work by He et al. and Zhu et al. has indicated that MUS can enhance the antibiotic effect on biofilms of* S. aureus* and* S. epidermidis *[18, 19]. Since micropores are observed on the biofilms after MUS intervention, they believe that MUS might increase the biofilm permeability and stimulate the transport of antibiotics through the biofilm. Obviously, there is a difference between biofilms and planktonic bacteria. The bacteria in biofilms are sequestered in layers of exopolymers, which protect them from normal levels of antimicrobial therapy [20]. Ultrasonication significantly increases the transport of antibiotics through the biofilm [21], thus increasing their lethality.

However, the barrier between planktonic bacteria and antibiotics is the bacterial cell wall. Neither He et al. nor Zhu et al. provide evidence of sonoporation of bacteria cell walls [18, 19]. By contrast, our study might prove the occurrence of sonoporation in* E. coli* by using electron microscopy (Figure 3). Partial disruption and higher thickness of the bacterial cell walls in the G_US_ and G_MUS_ may be an indication of sonoporation.

**Figure 5:**
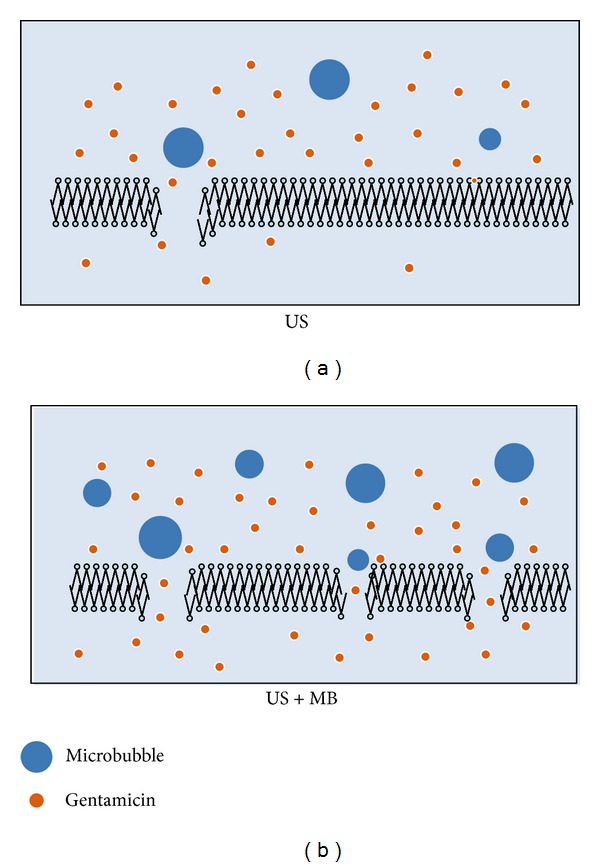
Nonthermal effect of stable cavitation by MUS. The mechanical effect of cavitating bubbles created pores in the cell membrane. This allowed gentamicin to enter the bacteria via passive diffusion. (a) In G_US_, there were sparse microbubbles and only a few gentamicin particles passed through cell membrane. (b) Addition of external microbubbles strengthened the cavitation, created more pores, and drove more drugs through bacterial cell membrane.

The physical stress caused by cavitation decreased the stability of the bacterial outer membrane, then the bacterial membrane loosened after ultrasonic irradiation, and, consistent with previous study [22], the cellular walls appeared to be thicker than normal. Only in the G_MUS_ group, discontinuity could be observed, a proof that MUS created a stronger cavitation than US alone. There was no significant difference regarding the mean thickness between the G_MUS_ and G_US_ groups, maybe because of the rapid decrease of the permeability [23]. As pores in the bacterial cell membranes reseal in the order of seconds after switching off the ultrasound [24], most of the small pores and disruption of cell walls had been resealed by the time the sample preparation for TEM was completed. However, we were able to observe some residual pores in the G_MUS_ group with TEM in our study. Pore formation might be the main mechanism for small impermeable molecules [25]. We believed that sonoporation of the cell membranes of* E. coli* could have occurred and that this was amplified by external microbubbles (Figure 5). In our study, MUS further reduced viable bacteria and induced more destruction of the bacterial cell walls. We assumed that external MB, acted as cavitation nuclei, exerted additional microstream and shear stress on cell membranes, and created more pores during sonication. Microbubbles amplified the biophysical effect of US and thus possibly improved the drug transport into the bacteria. Ultrasound and MUS did not kill bacteria without antibiotics. The reason for this inability may be that the size of the pores created by low-intensity ultrasound is large enough to deliver the drug but not severe enough to create fatal damage [26].

US is an inexpensive, easy to handle, and noninvasive tool. The level of shear stress is largely dependent on US parameters [27], and we can adjust it with an acoustic pressure between 100 Pa and 1000 Pa [28]. According to Williams and Pitt [8], there appears to be an intensity threshold (between 100 and 10 mW/cm^2^) below which ultrasound may not enhance the bactericidal effect of antibiotics. Pitt et al. establish that the viability of* E. coli* begins to decrease after 6 hours of sonication in combination with gentamicin [3]. An exposure to US irradiation with higher intensity and longer durations could accelerate the drug transport [5, 21]. However, it may cause skin damage [29]. As external microbubble could enhance the cavitation effect, lower intensity ultrasound with microbubble could be used to obtain equal efficacy and avoid tissue damage. Some studies have reported that low frequency US had a stronger bactericidal effect in the presence of antibiotics because the shear stress decreases as the frequency increases [30, 31]. In our model, we exposed* E. coli* solution to low frequency US at 100 mW/cm^2^ for 12 hours and dramatic improvement was observed. The encouraging results might be due to the US parameters of low frequency, relatively high intensity, and long duration; all these US parameters were within the range of clinical physical therapy [32]. The exposure time of 12 hours is too long for therapeutic purpose. Further experiments are needed to reach an optimum combination of parameters.

We acknowledged certain limitations in the present study. (i) The TEM images are useful to investigate cell morphology after sonoporation. However, to achieve real-time and direct evidence, ultrafast imaging and fluorescently labeled marker molecules may be better techniques to study the interaction of cavitating microbubbles with bacteria in suspension. (ii) The concentration of microbubble was set at 10% (*v/v*). Therefore, further studies are required to reveal the threshold below which the ultrasonic effect is no longer significant. (iii) The results of the* in vitro* study of planktonic bacteria cannot be transferred to human beings. (iv) The antimicrobial efficacy of the combination of MUS and antibiotic on bacteria and the involved cellular and molecular mechanisms deserve further investigation.

## 5. Conclusions

In conclusion, this research has demonstrated significant evidence that MUS, when combined with gentamicin, could further enhance the bactericidal effect and cause partial destruction of the bacterial cell wall. This technique may become a new treatment modality for bacterial infections.
